# Reproductive lifespan and cardiovascular health in postmenopausal women: a non-linear, behavior-driven association with cross-population validation

**DOI:** 10.3389/fmed.2026.1909845

**Published:** 2026-08-31

**Authors:** Jie Chen, Jia-yu Shi, Qian Guo, Shi-min Wu, Gu-quan Ma, Xiao-wei Zhao, Yong Tan, Ying-hao Pei

**Affiliations:** 1 Department of Gynecology, Jiangsu Province Hospital of Chinese Medicine, Affiliated Hospital of Nanjing University of Chinese Medicine, Nanjing, China; 2 Nanjing University of Chinese Medicine, Nanjing, China; 3 Department of Rheumatology, Jiangsu Province Hospital of Chinese Medicine, Affiliated Hospital of Nanjing University of Chinese Medicine, Nanjing, China; 4 Department of Gynecology, The People’s Hospital of Dazu, Chongqing, China; 5 Department of Intensive Care Unit, Jiangsu Province Hospital of Chinese Medicine, Affiliated Hospital of Nanjing University of Chinese Medicine, Nanjing, China

**Keywords:** cardiovascular health, Life’s Simple 7, mediation analysis, neutrophil-to-lymphocyte ratio, NHANES, non-linear association, reproductive lifespan

## Abstract

**Background:**

Reproductive lifespan (RLS), the interval from menarche to natural menopause, has been associated with cardiovascular disease (CVD) risk. However, most prior studies have assumed a linear relationship, and the potential non-linearity of this association, as well as the underlying behavioral and biological mechanisms, remain largely unexplored.

**Methods:**

This cross-sectional study included 1,673 naturally postmenopausal women (weighted *N* = 6,885,059) from eight NHANES cycles (1999–2010 and 2017–March 2020). Cardiovascular health was assessed using the American Heart Association’s Life’s Simple 7 (LS7) score. Restricted cubic spline and segmented non-linear analyses examined non-linearity. Mediation analyses evaluated indirect effects of alkaline phosphatase (ALP) and the neutrophil-to-lymphocyte ratio (NLR). Sensitivity analyses decomposed LS7 into behavioral and biological sub-scores. An independent cohort of 303 Chinese postmenopausal women was used to assess cross-population generalizability.

**Results:**

RLS exhibited a significant inverted U-shaped association with LS7 (P for non-linearity = 0.025). In the ascending segment (RLS ≤ 37 years), each additional year of RLS was associated with a higher LS7 score (β = 0.038, *P* = 0.048); beyond 37 years, the association became non-significant. NLR significantly mediated this association (P < 0.001; 8.8% mediated), whereas ALP showed no mediation. The non-linear association was driven entirely by the behavioral sub-score, with no contribution from the biological sub-score. The non-linear association was independently validated in the Chinese cohort (P for non-linearity = 0.014), and the sLS7 score differed significantly across RLS tertiles (5.71 vs. 5.69 vs. 4.94, *P* = 0.001).

**Conclusion:**

In naturally postmenopausal women, RLS showed an inverted U-shaped association with cardiovascular health, peaking at approximately 37 years. This association was partially mediated by NLR and driven entirely by behavioral components. These findings support incorporating reproductive history into cardiometabolic risk assessment and highlight behavioral interventions for women with shorter reproductive lifespans.

## 1 Introduction

Cardiovascular disease (CVD) remains the leading cause of death among women worldwide, accounting for approximately 35% of all female deaths annually (1). A striking feature of cardiovascular risk in women is its marked acceleration after menopause, suggesting that reproductive aging plays a pivotal role in the pathogenesis of cardiovascular disease (2). This observation has motivated growing interest in understanding how the timing and duration of reproductive events shape long-term cardiovascular health.

Reproductive lifespan (RLS), defined as the interval from menarche to natural menopause, represents the total duration of endogenous estrogen exposure across a woman’s lifetime (3, 4). Accumulating evidence from large-scale epidemiological studies, including several analyses of the National Health and Nutrition Examination Survey (NHANES), has demonstrated that a shorter RLS is associated with an elevated risk of cardiovascular events (5, 6) and mortality (7). However, the majority of these studies have assumed a linear relationship between RLS and cardiovascular outcomes. An analysis of NHANES data reported a non-linear, L-shaped association between RLS and cardiovascular mortality (7). Furthermore, a recent systematic review and meta-analysis demonstrated a J-shaped association between age at menarche, a key component of RLS, and cardiovascular events (8), providing additional evidence that reproductive factors may exhibit non-linear relationships with cardiovascular outcomes. These findings raise the possibility that similar non-linear patterns may exist for subclinical cardiovascular damage. Whether a similar non-linear pattern exists between RLS and subclinical cardiovascular damage remains largely unexplored. The NHANES dataset, with its comprehensive reproductive history questionnaire, laboratory measurements, and cardiovascular health metrics, offers a unique opportunity to investigate the RLS–CVH association in a nationally representative sample of US women.

Life’s Simple 7 (LS7), developed by the American Heart Association in 2010, provides a comprehensive assessment of cardiovascular health by integrating four health behaviors (smoking, diet, physical activity, and body mass index) and three health factors (blood pressure, total cholesterol, and fasting glucose) into a single composite score (9). Unlike clinical CVD events, which represent the end stage of a decades-long pathological process, the LS7 score captures subclinical cardiovascular damage at an earlier, potentially modifiable stage (10). This makes LS7 a particularly suitable outcome for investigating the subtle and cumulative effects of reproductive aging on the cardiovascular system. To our knowledge, the association between RLS and LS7 in naturally postmenopausal women has not been specifically examined, and the potential non-linearity of this relationship remains unexplored. The LS7 score, which integrates key metabolic indicators such as BMI, blood glucose, and lipid profiles, is a well-established predictor of cardiometabolic outcomes.

The biological mechanisms linking RLS to cardiovascular health are not fully understood. Chronic low-grade inflammation, a hallmark of estrogen deficiency after menopause (11), has been proposed as a key mediator. Two circulating biomarkers, alkaline phosphatase (ALP) and the neutrophil-to-lymphocyte ratio (NLR), were selected to represent distinct facets of this pathway. Several established mediators, including estrogen deficiency itself, endothelial dysfunction, and specific inflammatory cytokines, are either not directly measurable in large-scale epidemiological datasets such as NHANES or are subject to substantial measurement variability. We therefore focused on two biomarkers that are routinely measured, have well-characterized assay methods, and are supported by prior evidence linking them to both reproductive aging and cardiovascular outcomes. ALP, a marker of vascular calcification and bone turnover, has been associated with cardiovascular risk in postmenopausal women (12), which may partially mediate the RLS–cardiovascular health link. NLR, a systemic immune-inflammatory index, has been strongly linked to atherosclerotic cardiovascular events in large-scale analyses (13). Examining these two biomarkers in parallel offers an opportunity to disentangle the contributions of calcification-related versus immune-inflammatory pathways.

To address these gaps, we conducted this cross-sectional study using NHANES data to examine the RLS–LS7 association in naturally postmenopausal women, with particular attention to potential non-linearity. We also evaluated the mediating roles of ALP and NLR, and further decomposed LS7 into behavioral and biological sub-scores to identify which dimension primarily drove the observed association.

## 2 Materials and methods

### 2.1 Study design and data source

This cross-sectional study utilized data from the National Health and Nutrition Examination Survey (NHANES), a nationally representative survey of the non-institutionalized U.S. civilian population. Data were obtained from eight continuous NHANES cycles: 1999–2000, 2001–2002, 2003–2004, 2005–2006, 2007–2008, 2009–2010, 2017–2018, and the 2019–March 2020 pre-pandemic cycle. These data can be found at the National Center for Health Statistics (NCHS) NHANES website: https://wwwn.cdc.gov/nchs/nhanes/Default.aspx. NHANES was approved by the National Center for Health Statistics (NCHS) Ethics Review Board, and all participants provided written informed consent prior to data collection. The current study is a secondary analysis of publicly available, de-identified data and did not require additional ethical approval.

### 2.2 Study population

**FIGURE 1:**
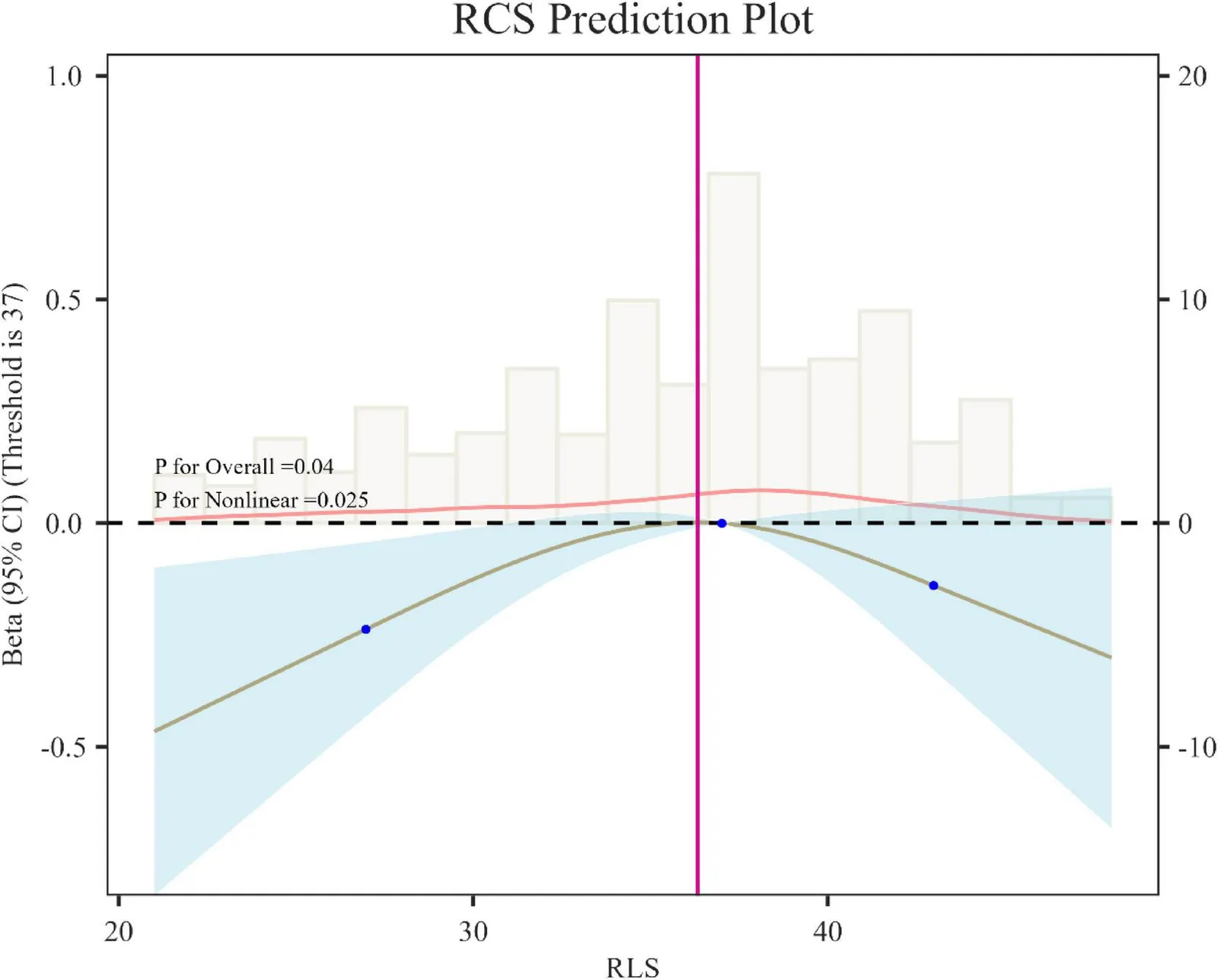
Association between reproductive lifespan and LS7 total score using restricted cubic spline analysis. Adjusted for age, race/ethnicity, education, marital status, poverty income ratio, smoking status, physical activity time, history of hypertension, history of diabetes, and history of kidney disease. The dashed line marks the inflection point at 37 years. P for non-linearity = 0.025.

From a total of 101,316 female respondents across the eight cycles, we applied the following exclusion criteria: (1) age < 40 years (*n* = 52,847 excluded), (2) not naturally postmenopausal (hysterectomy, bilateral oophorectomy, radiation/chemotherapy, or other medical reasons for amenorrhea; *n* = 31,486 excluded), (3) missing data on age at menarche or age at menopause, precluding calculation of RLS (*n* = 8,473 excluded), and (4) missing data on any of the seven LS7 components (*n* = 6,827 excluded). The final analytic sample comprised 1,673 naturally postmenopausal women (weighted *N* = 6,885,059). A detailed flow diagram of participant selection is provided in Figure 1.

### 2.3 Definition of RLS

RLS was defined as the interval between age at menarche and age at natural menopause. Age at menarche was assessed by the question, “How old were you when you had your first menstrual period?” Age at natural menopause was assessed by the question, “How old were you when you had your last menstrual period?” among women who reported natural menopause as the reason for cessation of periods. RLS was calculated as: RLS = age at natural menopause - age at menarche.

### 2.4 Cardiovascular health assessment: LS7

The primary outcome was the LS7 total score, a validated measure of subclinical cardiovascular damage. Secondary outcomes included individual CVD events. Cardiovascular health was assessed using the American Heart Association’s LS7 composite score (9), which comprises seven components: smoking, diet, physical activity, body mass index (BMI), blood pressure, total cholesterol, and fasting plasma glucose. Each component was classified as ideal (2 points), intermediate (1 point), or poor (0 points) according to established AHA criteria, yielding a total score ranging from 0 to 14.

Smoking status was derived from self-reported data. Dietary intake was assessed using a single 24-h dietary recall and operationalized as total daily sodium intake (mg/day). Total sodium was chosen as a pragmatic indicator because more comprehensive dietary quality indices (e.g., the Healthy Eating Index) were not consistently available across all survey cycles included.

Physical activity was quantified as total weekly minutes of moderate-to-vigorous activity. BMI was calculated from measured weight and height. Blood pressure was the average of up to three measurements. Total cholesterol and fasting plasma glucose were measured using standard enzymatic methods according to NHANES laboratory protocols (NCHS Laboratory Manual).^1^ For participants taking antihypertensive, antidiabetic, or lipid-lowering medications, the corresponding component was classified as “intermediate” rather than “ideal” if their measured values fell within the ideal range.

### 2.5 Circulating biomarkers

Two circulating biomarkers with distinct biological functions were examined as potential mediators. Alkaline phosphatase (ALP), a marker of vascular calcification and bone metabolism(12), was measured using a colorimetric enzymatic assay as part of the NHANES standard biochemistry panel. ALP was selected based on recent evidence linking it to the RLS–cardiovascular health association. The neutrophil-to-lymphocyte ratio (NLR), a systemic immune-inflammatory index, was calculated as the absolute neutrophil count divided by the absolute lymphocyte count. NLR was additionally analyzed after natural logarithmic transformation (logNLR) to better approximate normality.

### 2.6 CVD events

As secondary outcomes, CVD events were assessed by self-reported physician diagnosis of any of the following conditions: congestive heart failure, coronary heart disease, angina pectoris, myocardial infarction, and stroke. A composite CVD variable was defined as having at least one of these five conditions.

### 2.7 Covariates

Covariates were selected based on prior knowledge of potential confounders of the association between RLS and cardiovascular health. Demographic covariates included age, race/ethnicity, education level, marital status, and family poverty income ratio (PIR). Behavioral covariates included smoking status and physical activity time. Clinical covariates included self-reported history of hypertension, diabetes, and kidney disease.

### 2.8 Independent validation cohort and comparability with NHANES

To validate the primary findings from NHANES, an independent cohort of naturally postmenopausal women (*N* = 303) was recruited from Jiangsu Province Hospital of Chinese Medicine between December 2024 and July 2025. Inclusion and exclusion criteria were consistent with the NHANES analysis. A simplified LS7 score (sLS7, range 0–10) was used, comprising five components (smoking, BMI, blood pressure, total cholesterol, and fasting glucose), as data on physical activity and dietary sodium intake were not available in this clinical dataset. RLS groups were defined by tertiles, yielding three equal-sized groups of 101 participants each (T1, T2, and T3). Identical statistical methods were applied, including restricted cubic spline analysis, segmented non-linear fitting with the 37-year inflection point identified in NHANES, and behavioral/biological decomposition. All participants provided written informed consent, and the study was approved by the Ethics Committee of Jiangsu Province Hospital of Chinese Medicine (2024NL-111-02).

Although the NHANES sample is representative of the US population, the generalizability of findings to non-Western populations remains uncertain. We therefore selected a Chinese validation cohort to evaluate cross-population robustness. Chinese women differ substantially from US women in cardiovascular risk profiles, reproductive characteristics, and lifestyle patterns, making this cohort a particularly informative setting for external validation. Replication of the primary findings in this setting despite these differences would strengthen confidence in their cross-population generalizability.

The validation cohort was designed to match the NHANES cohort across key dimensions. Both cohorts included naturally postmenopausal women aged ≥ 40 years without an upper age limit. RLS was calculated identically as age at natural menopause minus age at menarche. Five of the seven LS7 components, smoking status, BMI, blood pressure, total cholesterol, and fasting glucose, were assessed using identical definitions and measurement protocols, and the same AHA LS7 classification criteria (ideal = 2, intermediate = 1, poor = 0) were applied in both cohorts. Laboratory values were measured in identical units (total cholesterol: mmol/L; fasting glucose: mmol/L; ALP: U/L; NLR: dimensionless). Two components, physical activity and dietary sodium intake, were unavailable in the clinical dataset and were therefore excluded from the simplified sLS7 score used for validation; this difference is acknowledged in the Limitations.

### 2.9 Statistical analysis

All analyses accounted for the complex multi-stage survey design of NHANES by applying appropriate sample weights, primary sampling units (PSU), and strata in accordance with NCHS analytical guidelines. For analyses combining multiple survey cycles, a new combined weight variable was created by dividing the original 2-year MEC examination weight by the number of cycles included. Baseline characteristics were summarized across RLS groups (Short: < 34 years; Medium: 34–42 years; Long: ≥ 43 years, based on tertile-derived cut-points) using means ± standard deviations for normally distributed continuous variables, medians with interquartile ranges for skewed continuous variables, and weighted percentages for categorical variables. P for trend across RLS groups was calculated using linear regression for continuous variables and Rao-Scott chi-square tests for categorical variables.

The association between RLS and LS7 total score was examined using three complementary approaches: (1) linear fitting with RLS as a continuous variable, (2) segmented non-linear fitting using restricted cubic splines (RCS) with three knots, and (3) categorical analysis using the RLS groups described above. Three hierarchical models were constructed: Model 1 adjusted for age and race; Model 2 additionally adjusted for socioeconomic and behavioral factors (education, marital status, PIR, smoking status, and physical activity time); and Model 3 further adjusted for clinical comorbidities (history of hypertension, diabetes, and kidney disease).

Mediation analysis was performed using the R package mediation to examine whether ALP, NLR, and logNLR mediated the association between RLS and LS7 total score. The average causal mediation effect (ACME), average direct effect (ADE), total effect, and proportion mediated were estimated. Confidence intervals for the ACME and ADE were obtained using 1,000 non-parametric bootstrap resamples. All mediation models were adjusted for Model 3 covariates. In exploratory analyses, mediation was also evaluated in strata defined by the 37-year inflection point.

Secondary analyses examined the association between RLS and CVD events using complex survey-weighted logistic regression, with composite CVD, heart failure, and angina as outcomes. A series of sensitivity analyses were conducted to assess the robustness of the findings: (S1) excluding participants with prevalent CVD (i.e., self-reported history of coronary heart disease, heart failure, angina, myocardial infarction, or stroke), which resulted in a reduced analytic sample of *n* = 974; (S2) excluding women with early menopause (age at menopause < 40 years); (S3) using the LS7 behavioral sub-score (sum of smoking, physical activity, diet, and BMI component scores; range 0–8) as the outcome; and (S4) using the LS7 biological sub-score (sum of blood pressure, total cholesterol, and fasting glucose component scores; range 0–6) as the outcome.

Statistical significance was set at a two-sided *P* < 0.05. All analyses were performed using R software (version 4.3.0) with the survey, rms, and mediation packages.

## 3 Results

### 3.1 Baseline characteristics

The weighted baseline characteristics of the study population, stratified by RLS groups, are presented in Table 1. A total of 6,885,059 (weighted) naturally postmenopausal women were included, with 2,261,337 (32.8%) in the short RLS group (<34 years), 3,780,207 (54.9%) in the medium RLS group (34–42 years), and 843,516 (12.3%) in the long RLS group ( ≥ 43 years). The overall mean age was 62.59 ± 9.85 years, and the mean RLS was 35.66 ± 6.22 years.

**TABLE 1:** Baseline characteristics of study participants by reproductive lifespan groups.

Characteristic	Overall (*N* = 6,885,059)	Short RLS ( < 34y) (*N* = 2,261,337)	Medium RLS (34–42y) (*N* = 3,780,207)	Long RLS ( ≥ 43y) (*N* = 843,516)	P for trend
Demographics					
Age, years	62.59 ± 9.85	59.86 ± 10.90	63.43 ± 9.06	66.10 ± 8.42	<0.001
PIR	3.40 (1.77, 5.00)	3.25 (1.63, 5.00)	3.51 (1.89, 5.00)	3.04 (1.65, 5.00)	0.103
Race, *N* (%)					0.123
— Non-Hispanic White	5,336,268 (77.51%)	1,704,925 (75.39%)	2,930,390 (77.52%)	700,952 (83.10%)	
— Non-Hispanic Black	589,841 (8.57%)	243,665 (10.78%)	282,760 (7.48%)	63,415 (7.52%)	
— Other	958,950 (13.93%)	312,747 (13.83%)	567,056 (15.00%)	79,149 (9.38%)	
Education, *N* (%)					0.330
— < High school	1,048,044 (15.22%)	401,981 (17.78%)	540,329 (14.30%)	105,734 (12.53%)	
— High school/GED	1,739,217 (25.26%)	575,753 (25.46%)	929,679 (24.59%)	233,785 (27.72%)	
— Some college/AA	1,996,568 (29.00%)	705,725 (31.21%)	1,086,749 (28.75%)	204,094 (24.20%)	
— College graduate+	2,101,230 (30.52%)	577,878 (25.55%)	1,223,449 (32.36%)	299,903 (35.55%)	
Marital status, *N* (%)					0.285
— Married/living with partner	4,055,363 (58.90%)	1,427,118 (63.11%)	2,173,986 (57.51%)	454,259 (53.85%)	
— Widowed/divorced/ separated	2,384,507 (34.63%)	668,023 (29.54%)	1,375,069 (36.38%)	341,415 (40.48%)	
— Never married/other	445,190 (6.47%)	166,195 (7.35%)	231,152 (6.11%)	47,843 (5.67%)	
Reproductive history					
Age at menarche, years	12.70 ± 1.70	12.84 ± 1.80	12.86 ± 1.55	11.61 ± 1.67	<0.001
Age at menopause, years	48.37 ± 6.21	41.04 ± 4.01	51.03 ± 2.66	56.06 ± 2.26	<0.001
Reproductive lifespan, years	35.66 ± 6.22	28.20 ± 3.50	38.17 ± 2.35	44.45 ± 1.78	<0.001
**LS7 total score** (0–14)	**7.68 ± 2.32**	**7.50 ± 2.42**	**7.88 ± 2.25**	**7.27 ± 2.27**	**0.005**
Other laboratory					
ALP, U/L	75.53 ± 25.22	75.25 ± 24.78	75.29 ± 25.64	77.32 ± 24.56	0.824
NLR	1.87 (1.42, 2.57)	1.83 (1.38, 2.53)	1.88 (1.43, 2.67)	1.81 (1.33, 2.33)	0.196
Medical history, N (%)					
Hypertension	2,248,512 (32.66%)	679,397 (30.04%)	1,249,318 (33.05%)	319,797 (37.91%)	0.272
Diabetes	222,411 (3.23%)	72,427 (3.20%)	131,882 (3.49%)	18,101 (2.15%)	0.748
Congestive heart failure	179,774 (2.61%)	101,063 (4.47%)	68,494 (1.81%)	10,217 (1.21%)	0.004
Coronary heart disease	270,603 (3.93%)	106,778 (4.72%)	118,564 (3.14%)	45,260 (5.37%)	0.560
Angina	160,671 (2.33%)	88,950 (3.93%)	62,380 (1.65%)	9,341 (1.11%)	0.019
Myocardial infarction	196,935 (2.86%)	83,126 (3.68%)	93,310 (2.47%)	20,499 (2.43%)	0.616
Stroke	195,860 (2.84%)	95,018 (4.20%)	86,432 (2.29%)	14,409 (1.71%)	0.071
Kidney disease	149,126 (2.17%)	43,475 (1.92%)	78,954 (2.09%)	26,698 (3.17%)	0.637

Data are presented as Mean ± SD, Median (Q1, Q3), or N (weighted %). P for trend calculated using linear regression or Rao-Scott chi-square test, accounting for complex survey design. Detailed LS7 component distributions are provided in Supplementary Table 1.

Across the three groups, women with longer RLS were significantly older (*P* < 0.001), reflecting the inherent mathematical coupling between RLS and chronological age. No significant differences were observed in race/ethnicity (*P* = 0.123), education level (*P* = 0.330), marital status (*P* = 0.285), or poverty income ratio (*P* = 0.103) across groups. Detailed distributions of the individual LS7 components by RLS groups are provided in Supplementary Table 1. Notably, while individual LS7 component scores did not differ significantly across groups (all *P* ≥ 0.051), the composite LS7 total score exhibited a significant inverted U-shaped pattern, peaking in the medium RLS group (7.88 ± 2.25) and declining in both the short RLS group (7.50 ± 2.42) and the long RLS group (7.27 ± 2.27; *P* = 0.005). This divergence, where many small, non-significant differences cumulate into a significant composite difference, validates the use of the LS7 composite as a more sensitive and holistic measure of cardiovascular health than its individual parts alone. The two inflammatory biomarkers, ALP (*P* = 0.824) and NLR (*P* = 0.196), did not differ significantly across RLS groups in unadjusted comparisons. Notably, the prevalence of congestive heart failure was highest in the short RLS group (4.47%) and lowest in the long RLS group (1.21%; *P* = 0.004). A similar pattern was observed for angina (3.93% vs. 1.65% vs. 1.11%; *P* = 0.019).

### 3.2 Association between RLS and LS7 total score

Next, we performed multivariable linear regression to examine the association between RLS and LS7 total score. As shown in Table 2, in the linear fitting approach, no significant association was observed in any model (fully adjusted Model 3: *P* = 0.364). Given that the restricted cubic spline analysis, as shown in Figure 1, revealed a significant non-linear relationship (P for non-linearity = 0.025) with an inflection point at approximately 37 years, we further applied segmented non-linear fitting. As shown in Table 2, in the ascending segment (RLS ≤ 37 years), each additional year of RLS was associated with a significantly higher LS7 score (β = 0.038, *P* = 0.048); in the plateau/descending segment (RLS > 37 years), no significant association was observed (β = -0.038, *P* = 0.104). In categorical analysis, neither the short RLS group (*P* = 0.712) nor the long RLS group (*P* = 0.626) differed significantly from the medium RLS reference group, confirming that the conventional categorical approach failed to capture the non-linear relationship.

**TABLE 2:** Multivariable-adjusted association between reproductive lifespan and LS7 total score using linear, segmented non-linear, and categorical approaches.

Exposure	Model 1		Model 2		Model 3	
	β (95% CI)	P	β (95% CI)	P	β (95% CI)	P
Linear fitting						
RLS (per 1 year)	0.02 (−0.01, 0.05)	0.217	0.01 (−0.01, 0.03)	0.372	0.01 (−0.01, 0.03)	0.364
Segmented non-linear fitting						
RLS ≤ 37y (ascending)	0.053 (0.001, 0.105)	0.045	0.049 (0.008, 0.091)	0.021	0.038 (0.000, 0.076)	0.048
RLS > 37y (plateau/descending)	−0.046 (−0.100, 0.008)	0.094	−0.050 (−0.099, −0.002)	0.043	−0.038 (−0.083, 0.008)	0.104
Categorical analysis						
Medium RLS (34–42y)	Ref	—	Ref	—	Ref	—
Short RLS ( < 34y)	−0.02 (−0.70, 0.65)	0.954	0.15 (−0.48, 0.79)	0.643	0.11 (−0.47, 0.70)	0.712
Long RLS ( ≥ 43y)	−0.88 (−1.91, 0.15)	0.095	−0.21 (−1.23, 0.80)	0.685	−0.24 (−1.20, 0.73)	0.626
P for trend	—	0.710	—	0.381	—	0.712

Model 1: Adjusted for age and race. Model 2: Additionally adjusted for education, marital status, PIR, smoking status (original variable), and physical activity time. Model 3: Additionally adjusted for history of hypertension, history of diabetes, and history of kidney disease. Reproductive lifespan was analyzed using three approaches: (1) linear fitting as a continuous variable, (2) segmented non-linear fitting using restricted cubic splines with 3 knots, which partitioned the association at the inflection point of approximately 37 years into an ascending segment (RLS ≤ 37y) and a plateau/descending segment (RLS > 37y), and (3) categorical analysis based on tertile-derived cut-points. RLS, reproductive lifespan; LS7, Life’s Simple 7; PIR, poverty income ratio.

### 3.3 Mediation analysis of circulating biomarkers

Then, we performed mediation analysis to evaluate whether ALP, NLR, and log-transformed NLR mediated the association between RLS and LS7 total score. As shown in Table 3, in the overall sample, both NLR (ACME = 0.001, *P* < 0.001; proportion mediated = 8.8%) and logNLR (ACME = 0.002, *P* < 0.001; proportion mediated = 15.6%) exhibited statistically significant indirect effects, whereas ALP showed no significant mediation (*P* = 0.996). In exploratory analyses stratified by the 37-year inflection point, the mediating effects of NLR and logNLR were attenuated and did not reach statistical significance in either the ascending segment (*P* > 0.05) or the plateau/descending segment (*P* > 0.05), suggesting that the inflammatory mediation was a weak but consistent effect distributed across the entire RLS range rather than a segment-specific phenomenon.

**TABLE 3:** Mediation analysis of circulating biomarkers in the association between reproductive lifespan and LS7 total score: overall and stratified by the inflection point.

Mediator	Segment	*N*	Total effect β (95% CI)	Direct effect (ADE) β (95% CI)	Indirect effect (ACME) β (95% CI)	Proportion mediated	P for ACME
ALP	Overall	1673	0.007 (−0.213, 0.238)	0.010 (−0.004, 0.025)	−0.003 (−0.234, 0.222)	0.976	0.996
	RLS ≤ 37y	943	0.013 (−0.715, 0.661)	0.029 (−0.001, 0.056)	−0.016 (−0.742, 0.638)	0.959	0.948
	RLS > 37y	730	−0.030 (−1.676, 1.739)	−0.025 (−0.096, 0.046)	−0.004 (−1.700, 1.704)	0.987	0.962
NLR	Overall	1673	0.010 (−0.010, 0.030)	0.008 (−0.004, 0.020)	0.001 (0.000, 0.002)	0.088	<0.001
	RLS ≤ 37y	943	0.027 (−0.005, 0.057)	0.026 (−0.006, 0.057)	0.001 (−0.001, 0.002)	0.011	0.460
	RLS > 37y	730	−0.028 (−0.091, 0.037)	−0.033 (−0.102, 0.037)	0.004 (−0.005, 0.013)	−0.116	0.356
logNLR	Overall	1673	0.009 (−0.021, 0.020)	0.008 (−0.004, 0.019)	0.002 (0.001, 0.002)	0.156	<0.001
	RLS ≤ 37y	943	0.027 (−0.005, 0.056)	0.025 (−0.007, 0.057)	0.001 (−0.002, 0.003)	0.045	0.090
	RLS > 37y	730	0.028 (−0.095, 0.041)	−0.033 (−0.105, 0.042)	0.005 (−0.007, 0.016)	−0.136	0.396

All models adjusted for age, race/ethnicity, education, marital status, PIR, smoking status (original variable), physical activity time, history of hypertension, history of diabetes, and history of kidney disease. The inflection point (37 years) was identified from segmented non-linear fitting in Table 2. 95% CIs for ACME and ADE were obtained using 1,000 bootstrap resamples. ALP, alkaline phosphatase; NLR, neutrophil-to-lymphocyte ratio; logNLR, natural log-transformed NLR; ADE, average direct effect; ACME, average causal mediation effect.

### 3.4 Association between RLS and cardiovascular disease events

Further, we examined the association between RLS and cardiovascular disease events using logistic regression. As shown in Table 4, in both linear and segmented non-linear analyses, RLS was not significantly associated with composite CVD (all *P* > 0.05) or heart failure (all *P* > 0.05). In the ascending segment (RLS ≤ 37 years), each additional year of RLS was associated with a borderline significant 10.6% reduction in angina risk (OR = 0.894, *P* = 0.053), a protective association that was absent in the plateau/descending segment (OR = 1.032, *P* = 0.712). In categorical analysis, a significant decreasing trend in angina prevalence was observed across RLS groups (P for trend = 0.003), although pairwise comparisons did not reach statistical significance. These findings suggest that the protective association of RLS with clinical cardiovascular events is modest and primarily confined to angina, consistent with the non-linear pattern observed for LS7.

**TABLE 4:** Association between reproductive lifespan and cardiovascular disease events: logistic regression analysis.

Exposure	Outcome	OR (95% CI)	P
Linear fitting			
RLS (per 1 year)	Composite CVD	1.047 (0.991, 1.106)	0.100
Segmented non-linear fitting			
RLS ≤ 37y	Composite CVD	1.019 (0.949, 1.093)	0.604
RLS > 37y	Composite CVD	1.031 (0.953, 1.116)	0.442
RLS ≤ 37y	Heart failure	0.945 (0.832, 1.074)	0.383
RLS > 37y	Heart failure	0.979 (0.822, 1.165)	0.808
RLS ≤ 37y	Angina	0.894 (0.798, 1.002)	0.053
RLS > 37y	Angina	1.032 (0.872, 1.222)	0.712
Categorical analysis			
Medium RLS (34–42y)	Composite CVD	Ref	—
Short RLS ( < 34y)	Composite CVD	0.682 (0.261, 1.783)	0.433
Long RLS ( ≥ 43y)	Composite CVD	1.066 (0.182, 6.253)	0.943
P for trend			0.369
Medium RLS (34–42y)	Heart failure	Ref	
Short RLS ( < 34y)	Heart failure	0.715 (0.171, 2.994)	0.646
Long RLS ( ≥ 43y)	Heart failure	0.616 (0.054, 7.016)	0.694
P for trend			0.247
Medium RLS (34–42y)	Angina	Ref	
Short RLS ( < 34y)	Angina	0.629 (0.138, 2.869)	0.549
Long RLS ( ≥ 43y)	Angina	0.738 (0.087, 6.270)	0.780
P for trend			0.003

All models adjusted for age, race/ethnicity, education, marital status, PIR, smoking status, physical activity time, history of hypertension, history of diabetes, and history of kidney disease. Complex survey design (PSU, Strata, MEC weights) accounted for. CVD, cardiovascular disease (composite of heart failure, coronary heart disease, angina, myocardial infarction, and stroke); RLS, reproductive lifespan; OR, odds ratio.

### 3.5 Sensitivity analyses

Finally, we conducted four sensitivity analyses to evaluate the robustness of the main findings. As shown in Supplementary Table 2, after excluding participants with prevalent CVD (S1) or early menopause (S2), the direction and magnitude of the association in the ascending segment remained consistent with the main analysis, although the reduced sample sizes led to attenuated statistical significance. Notably, when the LS7 total score was decomposed into behavioral and biological sub-scores, the non-linear association was entirely driven by the behavioral sub-score (S3: RLS ≤ 37 years, β = 0.029, *P* = 0.023; RLS > 37 years, β = -0.030, *P* = 0.028). In contrast, the biological sub-score showed no association with RLS in either segment (S4: both *P* > 0.4). These findings indicate that the relationship between RLS and cardiovascular health is primarily mediated through behavioral rather than direct biological pathways, and that the main results are robust to alternative sample restrictions.

### 3.6 Independent validation

The independent validation cohort, recruited from Jiangsu Province Hospital of Chinese Medicine between December 2024 and July 2025, comprised 303 naturally postmenopausal Chinese women (mean age 58.30 ± 7.53 years, mean RLS 37.89 ± 3.37 years). Baseline characteristics stratified by RLS tertiles are presented in Supplementary Table 3. Consistent with the NHANES findings, the sLS7 total score differed significantly across RLS tertiles (*P* = 0.001). Restricted cubic spline analysis confirmed a significant non-linear association between RLS and sLS7 (P for non-linearity = 0.014; Supplementary Figure 1). In segmented non-linear fitting, the ascending segment (RLS ≤ 37 years) showed a positive but non-significant association (β = 0.061, *P* = 0.143), while the descending segment (RLS > 37 years) was also non-significant (β = 0.047, *P* = 0.197; Supplementary Table 4). In the decomposition analysis, neither the behavioral sub-score nor the biological sub-score showed a significant association with RLS in either segment (Supplementary Table 5), although the biological sub-score contributed more to the overall sLS7 difference across tertiles (*P* = 0.013) than the behavioral sub-score (*P* = 0.014).

## 4 Discussion

This study extends prior work by demonstrating a non-linear, inverted U-shaped association between RLS and LS7 in naturally postmenopausal women, a pattern that challenges the linear assumptions adopted in most previous investigations. By decomposing LS7 into behavioral and biological sub-scores, we provide novel evidence that this association is driven primarily by health behaviors rather than by direct hormonal or metabolic effects, a distinction with direct implications for the design of preventive interventions. The cross-population validation in a Chinese cohort further demonstrates that this non-linear relationship is not restricted to US populations. From a clinical perspective, these findings suggest that RLS may serve as a complementary marker for cardiovascular risk stratification, identifying women with shorter reproductive lifespans who may benefit most from targeted behavioral interventions. From a public health perspective, the behavioral origin of this association highlights the importance of promoting healthy lifestyles, particularly smoking cessation, physical activity, and weight management, among women with early menopause or short reproductive lifespans, a group that is currently underrepresented in cardiovascular prevention guidelines.

This non-linear finding contrasts with the linear assumptions adopted in most prior investigations of RLS and cardiovascular outcomes (5, 6), which have uniformly modeled RLS as a continuous linear predictor. RCS analysis demonstrated that the RLS–LS7 relationship deviated significantly from linearity (P for non-linearity = 0.025), with LS7 scores peaking at approximately 37 years and declining thereafter. This pattern is consistent with a recent NHANES analysis reporting an L-shaped association between RLS and cardiovascular mortality (7), as well as with a recent meta-analysis documenting a J-shaped association between age at menarche and cardiovascular events (8). Collectively, these findings suggest that non-linear dynamics may characterize the relationship between reproductive factors and cardiovascular outcomes more broadly. The inflection point at approximately 37 years likely reflects the point at which the benefits of prolonged estrogen exposure reach a ceiling, beyond which chronological aging exerts a countervailing influence (14, 15). Supporting this, women in the long RLS group ( ≥ 43 years) were, on average, 6 years older than those in the short RLS group ( < 34 years).

A descending trend beyond 37 years was non-significant in the fully adjusted model (*P* = 0.104) and requires cautious interpretation; if confirmed, it may reflect survivor effects or age-related comorbidities. At present, the data primarily support an ascending benefit that plateaus around 37 years.

NLR significantly mediated the RLS–LS7 association, consistent with the hypothesis that chronic low-grade inflammation links reproductive aging to cardiovascular health (11, 16). After menopause, declining estrogen levels are associated with elevated pro-inflammatory cytokines and a shift toward a more inflammatory immune profile (11, 16). The stronger mediating effect of logNLR likely reflects improved normality. In contrast, ALP showed no significant mediation (*P* = 0.996). Although ALP has been associated with cardiovascular risk in postmenopausal women through bone turnover and vascular calcification (17), its lack of mediation may be attributable to differences in the cardiovascular health metric, study population, or more comprehensive adjustment for behavioral covariates. Moreover, ALP is a nonspecific enzyme with hepatic, bone, and vascular origins, and may be elevated in non-cardiovascular conditions such as liver disease, biliary obstruction, bone disorders, and vitamin D deficiency (18). These conditions were not specifically excluded from our analysis, which may have introduced residual confounding and attenuated any specific mediating effect operating through vascular calcification pathways. This limitation may partially explain why ALP did not significantly mediate the RLS–LS7 association in our study, in contrast to the significant mediation observed for NLR, which is a more specific marker of systemic inflammation.

Decomposition of the LS7 score revealed that the non-linear association was driven entirely by the behavioral sub-score (ascending: *P* = 0.023; descending: *P* = 0.028), with no contribution from the biological sub-score (both *P* > 0.4). This dissociation suggests that RLS influences cardiovascular health primarily through health behaviors rather than direct hormonal effects on physiological parameters (9), consistent with the foundational premise of the LS7 framework that behavioral factors are central to cardiovascular health. Women with longer RLS may adopt healthier lifestyles, or estrogen-mediated neurobiological effects may promote behaviors with lasting postmenopausal benefits (15, 19). Although our cross-sectional design cannot establish causality, these findings provide a compelling rationale for longitudinal studies.

In secondary analyses, longer RLS was associated with a borderline reduced angina risk in the ascending segment (OR = 0.894, *P* = 0.053; P for trend = 0.003), consistent with the non-linear pattern observed for LS7 (5, 7, 20). This signal may reflect angina’s sensitivity to microvascular dysfunction linked to estrogen deficiency (5, 21). The absence of an association with heart failure after adjustment, despite a significant unadjusted difference (*P* = 0.004), suggests that age and other risk factors largely account for this crude association.

Several limitations should be acknowledged. First, the cross-sectional design cannot establish causality. Second, self-reported RLS and CVD events are subject to recall bias. Third, dietary quality was assessed using sodium intake rather than a comprehensive index. Fourth, hormone replacement therapy data were unavailable. Fifth, the NHANES population was restricted to US women. Sixth, the validation cohort was relatively small (*N* = 303), recruited from a single Chinese center, and used a simplified LS7 score lacking physical activity and dietary components. Furthermore, population differences between the US NHANES cohort and the Chinese validation cohort, including differences in cardiovascular risk profiles, reproductive characteristics, and lifestyle patterns, may limit the direct comparability of the two samples. Seventh, ALP and NLR represent only a narrow window into the complex biology linking reproductive aging to cardiovascular health. ALP, in particular, is a nonspecific enzyme that may be elevated in hepatic, biliary, and bone disorders unrelated to cardiovascular risk; these conditions were not specifically excluded from the analysis and may have contributed to residual confounding in the mediation analysis.

In conclusion, in this nationally representative sample of naturally postmenopausal US women, RLS exhibited a significant non-linear association with cardiovascular health, characterized by an ascending phase up to approximately 37 years followed by a plateau. This association was partially mediated by NLR, a systemic immune-inflammatory index, and was driven entirely by behavioral rather than biological components of the LS7 score. These findings underscore the importance of moving beyond linear assumptions in reproductive epidemiology and highlight health behaviors as a potentially modifiable pathway linking reproductive aging to cardiovascular health. Future longitudinal studies are needed to confirm these findings and to evaluate whether behavioral interventions targeted at women with shorter RLS can improve cardiovascular outcomes.

## Data Availability

The raw data supporting the conclusions of this article will be made available by the authors, without undue reservation.

## Footnotes

1
https://wwwn.cdc.gov/nchs/nhanes/continuousnhanes/labmethods.aspx
