# Supplementary material for: Reproductive lifespan and cardiovascular health in postmenopausal women: a non-linear, behavior-driven association with cross-population validation
Source: Front Med (Lausanne). 2026 Aug 31;13:1909845. doi: 10.3389/fmed.2026.1909845 (PMC13572729; doi:10.3389/fmed.2026.1909845)
Supplement: Supplementary file 1 [file Table_1.docx]

**Table S1. Distribution of Individual LS7 Components by Reproductive Lifespan Groups**

| **LS7 Component** | **Overall** | **Short RLS** | **Medium RLS** | **Long RLS** | **P** |
| --- | --- | --- | --- | --- | --- |
| **Health Behaviors** |  |  |  |  |  |
| Smoking score, N (%) |  |  |  |  | 0.097 |
| — 0 (Poor) | 894,146 (12.99%) | 382,168 (16.90%) | 374,642 (9.91%) | 137,335 (16.28%) |  |
| — 1 (Intermediate) | 1,757,487 (25.53%) | 532,282 (23.54%) | 971,799 (25.71%) | 253,407 (30.04%) |  |
| — 2 (Ideal) | 4,233,426 (61.49%) | 1,346,887 (59.56%) | 2,433,766 (64.38%) | 452,774 (53.68%) |  |
| Physical activity, min/week | 21.00 (0.00, 180.00) | 30.00 (0.00, 180.00) | 28.00 (0.00, 180.00) | 0.00 (0.00, 100.00) | 0.073 |
| Physical activity score, N (%) |  |  |  |  | 0.247 |
| — 0 (Poor) | 3,203,491 (46.53%) | 1,093,059 (48.34%) | 1,667,321 (44.11%) | 443,110 (52.53%) |  |
| — 1 (Intermediate) | 1,710,052 (24.84%) | 506,752 (22.41%) | 976,485 (25.83%) | 226,815 (26.89%) |  |
| — 2 (Ideal) | 1,971,517 (28.63%) | 661,526 (29.25%) | 1,136,401 (30.06%) | 173,590 (20.58%) |  |
| Dietary sodium intake, mg/day | 1,552 (1,173, 2,050) | 1,595 (1,163, 2,179) | 1,531 (1,169, 2,015) | 1,560 (1,188, 1,902) | 0.543 |
| Diet (sodium) score, N (%) |  |  |  |  | 0.051 |
| — 0 (Poor) | 2,215,115 (32.17%) | 876,708 (38.77%) | 1,124,385 (29.74%) | 214,022 (25.37%) |  |
| — 1 (Intermediate) | 2,722,136 (39.54%) | 773,853 (34.22%) | 1,537,387 (40.67%) | 410,896 (48.71%) |  |
| — 2 (Ideal) | 1,947,808 (28.29%) | 610,776 (27.01%) | 1,118,434 (29.59%) | 218,598 (25.92%) |  |
| BMI, kg/m² | 28.39 ± 6.51 | 28.99 ± 7.15 | 27.97 ± 6.18 | 28.64 ± 6.02 | 0.105 |
| BMI score, N (%) |  |  |  |  | 0.280 |
| — 0 (Poor) | 2,209,477 (32.09%) | 805,927 (35.64%) | 1,096,327 (29.00%) | 307,223 (36.42%) |  |
| — 1 (Intermediate) | 2,302,349 (33.44%) | 719,885 (31.83%) | 1,302,867 (34.47%) | 279,596 (33.15%) |  |
| — 2 (Ideal) | 2,373,233 (34.47%) | 735,525 (32.53%) | 1,381,012 (36.53%) | 256,696 (30.43%) |  |
| **Health Factors** |  |  |  |  |  |
| SBP, mmHg | 127.14 ± 19.41 | 125.72 ± 18.51 | 127.15 ± 19.29 | 130.86 ± 21.76 | 0.093 |
| DBP, mmHg | 71.32 ± 11.40 | 71.74 ± 11.39 | 70.87 ± 11.00 | 72.24 ± 13.07 | 0.642 |
| Blood pressure score, N (%) |  |  |  |  | 0.594 |
| — 0 (Poor) | 1,581,810 (22.97%) | 480,525 (21.25%) | 856,951 (22.67%) | 244,334 (28.97%) |  |
| —1 (Intermediate) | 3,345,997 (48.60%) | 1,095,151 (48.43%) | 1,876,992 (49.65%) | 373,854 (44.32%) |  |
| — 2 (Ideal) | 1,957,252 (28.43%) | 685,662 (30.32%) | 1,046,263 (27.68%) | 225,327 (26.71%) |  |
| Total cholesterol, mmol/L | 5.38 ± 1.06 | 5.34 ± 1.12 | 5.40 ± 1.01 | 5.42 ± 1.07 | 0.569 |
| Total cholesterol score, N (%) |  |  |  |  | 0.903 |
| — 0 (Poor) | 1,438,642 (20.90%) | 448,762 (19.85%) | 821,442 (21.73%) | 168,438 (19.97%) |  |
| — 1 (Intermediate) | 3,895,489 (56.58%) | 1,296,901 (57.35%) | 2,097,898 (55.50%) | 500,690 (59.36%) |  |
| — 2 (Ideal) | 1,550,928 (22.53%) | 515,674 (22.80%) | 860,866 (22.77%) | 174,388 (20.67%) |  |
| Fasting glucose, mg/dL | 101.00 (94.00, 111.00) | 101.00 (94.00, 111.00) | 101.00 (94.00, 110.00) | 101.00 (94.00, 111.00) | 0.800 |
| Fasting glucose score, N (%) |  |  |  |  | 0.803 |
| — 0 (Poor) | 800,042 (11.62%) | 287,633 (12.72%) | 394,533 (10.44%) | 117,877 (13.97%) |  |
| — 1 (Intermediate) | 3,072,517 (44.63%) | 1,015,649 (44.91%) | 1,688,782 (44.67%) | 368,085 (43.64%) |  |
| — 2 (Ideal) | 3,012,500 (43.75%) | 958,055 (42.37%) | 1,696,892 (44.89%) | 357,554 (42.39%) |  |
| HDL, mmol/L | 1.59 ± 0.46 | 1.56 ± 0.48 | 1.62 ± 0.46 | 1.54 ± 0.40 | 0.051 |

**Notes:** Data are presented as Mean ± SD for normally distributed continuous variables, Median (Q1, Q3) for skewed continuous variables, and N (weighted %) for categorical variables. Normally distributed continuous variables include: BMI, SBP, DBP, total cholesterol, and HDL. Skewed continuous variables include: physical activity, dietary sodium intake, and fasting glucose. P for trend across reproductive lifespan groups was calculated using linear regression (continuous variables) or Rao-Scott chi-square test (categorical variables), accounting for the complex survey design (PSU, Strata, and MEC weights). Reproductive lifespan groups were defined as: Short RLS (<34 years), Medium RLS (34–42 years), and Long RLS (≥43 years), based on tertile-derived cut-points. Dietary sodium intake values were multiplied by 10 to approximate total daily sodium intake in mg. RLS, reproductive lifespan; BMI, body mass index; SBP, systolic blood pressure; DBP, diastolic blood pressure; HDL, high-density lipoprotein cholesterol; LS7, Life's Simple 7.

**Table S2. Sensitivity Analyses: Association Between Reproductive Lifespan and LS7 Total Score Under Alternative Model Specifications**

| **Analysis** | **Sample** | **N** | **Exposure** | **β (95% CI)** | **P** |
| --- | --- | --- | --- | --- | --- |
| **Main analysis (Table 2, Model 3)** | Full sample | 1,673 | RLS (per 1 year) | 0.010 (-0.010, 0.030) | 0.364 |
|  |  |  | RLS ≤ 37y | 0.038 (0.000, 0.076) | 0.048 |
|  |  |  | RLS > 37y | -0.038 (-0.083, 0.008) | 0.104 |
| **S1: Excluding prevalent CVD** | CVD-free only | 974 | RLS (per 1 year) | 0.027 (-0.001, 0.054) | 0.053 |
|  |  |  | RLS ≤ 37y | 0.038 (-0.021 0.076) | 0.261 |
|  |  |  | RLS > 37y | -0.001 (-0.057, 0.054) | 0.963 |
| **S2: Excluding early menopause (<40y)** |  | 1521 | RLS (per 1 year) | 0.005 (-0.025, 0.034) | 0.763 |
|  |  |  | RLS ≤ 37y | 0.045 (-0.008, 0.098) | 0.095 |
|  |  |  | RLS > 37y | -0.053 (-0.109, 0.004) | 0.068 |
| **S3: LS7 behavioral sub-score as outcome** | Full sample | 1,673 | RLS (per 1 year) | 0.005 (-0.006, 0.016) | 0.337 |
|  |  |  | RLS ≤ 37y | 0.029 (0.004, 0.054) | 0.023 |
|  |  |  | RLS > 37y | -0.030 (-0.057, -0.003) | 0.028 |
| **S4: LS7 biological sub-score** | Full sample | 1,673 | RLS (per 1 year) | -0.004 (-0.016, 0.009) | 0.576 |
|  |  |  | RLS ≤ 37y | -0.009 (-0.031, 0.014) | 0.458 |
|  |  |  | RLS > 37y | 0.006 (-0.020, 0.032) | 0.628 |

**Notes**: All models adjusted for age, race/ethnicity, education, marital status, PIR, smoking status (original variable), physical activity time, history of hypertension, history of diabetes, and history of kidney disease, unless otherwise noted. Complex survey design (PSU, Strata, MEC weights) was accounted for in all models. S1: Excludes participants with self-reported history of coronary heart disease, heart failure, angina, myocardial infarction, or stroke. The variable "history of hypertension" was excluded from S1 models due to zero prevalence in the CVD-free subset. S2: Excludes women with age at menopause < 40 years. S3: Outcome is LS7 behavioral sub-score, calculated as the sum of smoking, physical activity, diet (sodium), and BMI component scores (range 0–8). S4: Outcome is LS7 biological sub-score, calculated as the sum of blood pressure, total cholesterol, and fasting glucose component scores (range 0–6). RLS, reproductive lifespan; LS7, Life's Simple 7; CVD, cardiovascular disease; PIR, poverty income ratio; BMI, body mass index.

**Table S3. Baseline Characteristics of the Independent Validation Cohort by RLS Tertiles**

| **Characteristic** | **Overall (N=303)** | **Short RLS (T1) (N=101)** | **Medium RLS (T2) (N=101)** | **Long RLS (T3) (N=101)** | **P for trend** |
| --- | --- | --- | --- | --- | --- |
| **Demographics** |  |  |  |  |  |
| Age, years | 58.30 ± 7.53 | 55.62 ± 7.60 | 58.00 ± 7.26 | 61.29 ± 6.66 | <0.001 |
| RLS, years | 37.89 ± 3.37 | 34.23 ± 2.26 | 38.06 ± 0.76 | 41.39 ± 1.61 | <0.001 |
| Marital Status, N (%) |  |  |  |  | 0.134 |
| — Married | 290 (95.71%) | 100 (99.01%) | 95 (94.06%) | 95 (94.06%) |  |
| — Widowed | 13 (4.29%) | 1 (0.99%) | 6 (5.94%) | 6 (5.94%) |  |
| Education, N (%) |  |  |  |  | 0.211 |
| —Middle school or below | 222 (73.27%) | 67 (66.34%) | 73 (72.28%) | 82 (81.19%) |  |
| —High school or college | 62 (20.46%) | 26 (25.74%) | 21 (20.79%) | 15 (14.85%) |  |
| —Bachelor's degree or above | 19 (6.27%) | 8 (7.92%) | 7 (6.93%) | 4 (3.96%) |  |
| **Cardiovascular Health** |  |  |  |  |  |
| sLS7 Total Score (0–10) | 5.45 ± 1.61 | 5.71 ± 1.47 | 5.69 ± 1.60 | 4.94 ± 1.65 | **0.001** |
| Behavioral sub-score (0–4) | 1.59 ± 0.56 | 1.67 ± 0.49 | 1.64 ± 0.52 | 1.45 ± 0.62 | **0.014** |
| Biological sub-score (0–6) | 3.86 ± 1.41 | 4.04 ± 1.33 | 4.05 ± 1.42 | 3.50 ± 1.43 | **0.013** |
| **LS5 Components** |  |  |  |  |  |
| BMI, kg/m² | 24.18 ± 3.07 | 23.63 ± 2.77 | 24.06 ± 2.76 | 24.86 ± 3.51 | **0.022** |
| SBP, mmHg | 128.19 ± 17.29 | 124.39 ± 15.73 | 129.64 ± 19.31 | 130.54 ± 16.15 | **0.015** |
| DBP, mmHg | 80.53 ± 10.46 | 78.24 ± 9.53 | 81.50 ± 11.98 | 81.86 ± 9.40 | **0.016** |
| Total cholesterol, mmol/L | 4.95 ± 0.91 | 4.87 ± 0.90 | 4.88 ± 0.83 | 5.11 ± 0.99 | 0.104 |
| Fasting glucose, mmol/L | 5.54 ± 1.42 | 5.51 ± 0.94 | 5.34 ± 0.87 | 5.76 ± 2.09 | 0.315 |
| HDL, mmol/L | 1.39 ± 0.29 | 1.39 ± 0.28 | 1.40 ± 0.28 | 1.39 ± 0.31 | 0.896 |
| **Laboratory Biomarkers** |  |  |  |  |  |
| ALP, U/L | 83.65 ± 24.80 | 80.40 ± 24.96 | 82.45 ± 22.00 | 88.15 ± 26.82 | 0.094 |
| NLR | 2.60 ± 1.65 | 2.58 ± 1.40 | 2.65 ± 1.83 | 2.58 ± 1.71 | 0.685 |
| **Medical History, N (%)** |  |  |  |  |  |
| Hypertension | 77 (25.41%) | 17 (16.83%) | 26 (25.74%) | 34 (33.66%) | 0.023 |
| Antihypertensive medication | 74 (24.42%) | 16 (15.84%) | 25 (24.75%) | 33 (32.67%) | 0.021 |
| Diabetes | 33 (10.89%) | 10 (9.90%) | 11 (10.89%) | 12 (11.88%) | 0.903 |
| Antidiabetic medication | 24 (7.92%) | 5 (4.95%) | 10 (9.90%) | 9 (8.91%) | 0.387 |
| Heart failure | 8 (2.64%) | 3 (2.97%) | 0 (0.00%) | 5 (4.95%) | 0.087 |
| Coronary heart disease | 5 (1.65%) | 2 (1.98%) | 1 (0.99%) | 2 (1.98%) | 0.816 |
| Stroke | 14 (4.62%) | 3 (2.97%) | 7 (6.93%) | 4 (3.96%) | 0.378 |
| Renal failure | 4 (1.32%) | 0 (0.00%) | 1 (0.99%) | 3 (2.97%) | 0.170 |

**Notes**: Data are presented as Mean ± SD for continuous variables and N (%) for categorical variables. P for trend across RLS tertiles was calculated using linear regression (continuous variables) or chi-square test (categorical variables). RLS tertile cut-points: T1 <35.3 years, T2 35.3–39.6 years, T3 >39.6 years. The sLS7 score (range 0–10) comprised smoking, BMI, blood pressure, total cholesterol, and fasting glucose. RLS, reproductive lifespan; sLS7, simplified Life's Simple 7; BMI, body mass index; SBP, systolic blood pressure; DBP, diastolic blood pressure; HDL, high-density lipoprotein cholesterol; ALP, alkaline phosphatase; NLR, neutrophil-to-lymphocyte ratio.

**Table S4. Association Between Reproductive Lifespan and sLS7: Independent Validation (N = 303)**

| **Exposure** | **β (95% CI)** | **P** |
| --- | --- | --- |
| RLS (per 1 year) — Linear fitting | -0.005 (-0.056, 0.046) | 0.846 |
| RLS ≤ 37y (ascending segment) | 0.061 (-0.021, 0.143) | 0.143 |
| RLS > 37y (descending segment) | 0.047 (-0.025, 0.119) | 0.197 |
| P for non-linearity (RCS) | — | **0.0135** |

**Notes**: All models were adjusted for age, education, marital status, hypertension, diabetes, and renal failure. RLS was analyzed using two approaches: (1) linear fitting as a continuous variable, and (2) segmented non-linear fitting, which partitioned the association at the inflection point of 37 years into an ascending segment (RLS ≤ 37 years) and a descending segment (RLS > 37 years). P for non-linearity was obtained from restricted cubic spline analysis with 3 knots, comparing the RCS model with a linear model using ANOVA. RLS, reproductive lifespan; sLS7, simplified Life's Simple 7; RCS, restricted cubic spline.

**Table S5. Behavioral vs Biological Sub-scores in the Independent Validation Cohort (N = 303)**

| **Outcome** | **RLS ≤ 37y**  **β (95% CI)** | **P** | **RLS > 37y**  **β (95% CI)** | **P** |
| --- | --- | --- | --- | --- |
| Behavioral sub-score  (0–4) | 0.005 (-0.028, 0.037) | 0.777 | 0.002 (-0.027, 0.030) | 0.910 |
| Biological sub-score  (0–6) | 0.057 (-0.015, 0.128) | 0.122 | 0.046 (-0.017, 0.108) | 0.154 |

**Notes**: Behavioral sub-score comprised smoking and BMI components (range 0–4). Biological sub-score comprised blood pressure, total cholesterol, and fasting glucose components (range 0–6). All models adjusted for age, Education, Marital_Status, HBP, DM, and Renal_failure. RLS, reproductive lifespan.

**Figure S1. Restricted Cubic Spline Analysis of RLS and sLS7 in the Independent Validation Cohort**
